# Human Keratinocytes Cultured on Collagen Matrix Used as an Experimental Burn Model

**Published:** 2007-10-30

**Authors:** Christiane S. Sobral, Alfredo Gragnani, Xudong Cao, Jeffrey R. Morgan, Lydia Masako Ferreira

**Affiliations:** Skin Cell Culture Laboratory, Division of Plastic Surgery, Federal University of Sao Paulo (UNIFESP), Sao Paulo, Brazil; Department of Molecular Pharmacology, Physiology and Biotechnology, Brown University, Providence, RI; Division of Plastic Surgery of Federal University of Sao Paulo (UNIFESP), Sao Paulo, Brazil

## Abstract

**Background:** In experimental models in vivo, it is difficult to characterize the effect of thermal burns on epidermal keratinocytes. Since the response to thermal injury involves several systemic mechanisms, especially because of the stimulus to coagulation and inflammatory cascades, it becomes hard to evaluate the specific effect of thermal burns on keratinocytes. The aim of this study is to propose the use of human keratinocytes cultured on collagen matrix as an in vitro experimental burn model. **Methods:** Human keratinocytes derived from neonatal foreskins were isolated and cultured following standard methods. All experiments used the same keratinocyte lineage and were carried out in triplicate. Initially, gels of collagen and Matrigel were prepared. For each gel, 2 × 10^6^ keratinocytes were seeded and cultured to form stratified epithelia. Following, burn wounds were induced at 170°C. **Results:** Keratinocytes were cultured on collagen-coated Millicell membranes. Stratified epithelia were formed and burned on the seventh day after the cultures were raised to the air-liquid interface. The burn procedure is reproducible and can be easily executed. **Conclusion:** The proposed model can be used to study the effects of induced burn wounds on keratinocytes in a specific way.

The integrity of the epidermal barrier is one of the means by which the skin protects the body. In the situations when this barrier is compromised, as in major burns or ulcerations, it becomes necessary to close these wounds.^1^

The colonization and infection of the raw surface occur in a short period after the epidermal barrier is disrupted. The excision and early coverage of the wound with a permanent skin substitute, autografts, or cultured keratinocytes when donor sites are scarce, is the treatment that gives the best results for full-thickness burns, decreasing morbidity, mortality, and complication rates. However, it is impossible to immediately cover lesions in infected surface areas.^2^, ^3^

The establishment of an efficient protocol for keratinocyte culture has made its production possible by industrial laboratories and the clinical use of simple cultured keratinocyte sheets in burn patients. Burn treatment units that have this therapeutic tool started to use these cultured epithelial sheets in the treatment of the burn patient.^4^, ^5^

In mouse or pig experimental models in vivo, it is difficult to characterize the effect of thermal burns on epidermal keratinocytes in a separate way, since there are many variables involved. Because of the systemic response to thermal injuries, especially by the stimulus to coagulation and inflammatory cascades, it becomes hard to evaluate only the effect on keratinocytes produced by a direct stimulus.

The aim of this study is to propose the use of human keratinocytes cultured on collagen matrix as an in vitro experimental burn model.

## METHODS

### Keratinocyte culture

Normal human keratinocytes derived from neonatal foreskins were isolated and cultured following standard methods^6^ adapted in our laboratory,^7^ with some changes as described below. The same keratinocyte lineage was used in each experiment. All experiments were done in triplicate.

A skin fragment was placed in a 60-mm culture dish and cut into smaller fragments of about 0.5 cm^3^ each. These fragments were placed in a tube containing 30 mL of dispase solution (Boehringer Mannheim cat. No. 165859) and incubated overnight at 4°C for 15 hours.

In the next morning, the skin fragments were removed from the tube and placed in a 100-mm Petri dish. Next, the skin was washed with versene solution.

Just after, using 2 delicate clamps, each skin fragment had the epidermis peeled off from the dermis. The epidermis was placed in a 15-mL sterile tube containing 5 mL of 0.25% trypsin solution, and incubated for 10 minutes at 37°C under continuous agitation.

The supernatant was transferred to another 50-mL sterile tube and centrifuged for 5 minutes at 800 rpm. The remaining protocol follows the Laboratory Standard.^7^

### Collagen matrices

Millicell membranes (Millipore cat. No. PICMORG 50) were used in this stage of the experiment. Collagen type I (Vitrogen Collagen Corporation, Palo Alto, Calif) was used in the ratio of 8:1 with 10 × PBS (phosphate buffer solution), pH 7.4. The total solution volume was prepared in accordance with the number of matrices. A 4-mL collagen solution was produced to prepare 10 Millicell membranes, each containing 400 μL of collagen solution. After that, the pH of the solution was adjusted to 7.4.

Aliquots of Matrigel basement membrane matrix (BD Biosciences cat. No. 354234) were removed from the freezer and kept in ice for 2 hours. Ten aliquots of solution, each consisting of 400 μL of collagen, 400 μL of Matrigel, and 80 μL of 0.1% glutaraldehyde were placed in tubes.

Immediately after the mixing, 880 μL of solution was placed on each Millicell membrane (0.4 μm pore size). The set consisting of Millicell membrane, collagen, Matrigel, and glutaraldehyde was called matrix, receiving this designation after its production. The matrices were then placed in 35-mm Petri dishes and covered. After that, the 35-mm Petri dishes were placed in 150-mm plates, covered and incubated at 4°C for 12 hours.

After this period, the plates were placed inside an incubator at 37°C for 2 hours and 6 mL of 2% glycin solution was added. Each matrix was removed from the 150-mm plate and 35-mm Petri dish and transferred into another 150-mm sterile plate. Again, the solution was added to the plates, which were left in the incubator for 2 more hours. After this process, the glycine was aspirated and the matrices were kept soaked in 30 mL of phosphate buffer solution, in the incubator, until used.

### Keratinocyte seeding on matrices

Fourth passage keratinocytes at 80% confluence in a 175-cm^2^ flask were prepared for the seeding into the collagen matrices. The keratinocytes were subjected to the standard serial passage procedure. Each matrix was seeded with 2 × 10^6^ keratinocytes. At the time of seeding, the keratinocytes were resuspended in 0.1 mL of aprotinin solution, added to 0.9 mL of seeding medium (10:90, vol/vol), totaling a volume of 1 mL. Currie et al (2003) used a model of wound healing to evaluate the effect of fibrin glue as a dermal substitute.^8^

### The air-liquid interface

The keratinocytes were cultured on the collagen matrices at the air-liquid interface following the standard protocol.^7^, ^9^

After the cultures were raised to the air-liquid interface, the supplementation of the media was carried out by adding 1.8 mL culture medium around the Millicell membrane; no medium was added over the keratinocytes. The burn procedure took place on the seventh day after the cultures were raised to the air-liquid interface or the tenth day after the keratinocyte seeding.

## RESULTS

### Experimentally induced burn wounds

The culture medium of each plate was incubated for 24 hours before the beginning of the burn experiment. Immediately before the experiment, the medium was aspirated and stored. After that, each plate was transferred to a 35-mm Petri dish without culture medium and burned.

A constant-temperature hot plate at 170°C was used for the experiment; stainless steel rods were placed over it until a thermal balance was achieved. The stainless steel rods were used to burn the matrices and had predefined dimensions and mass.

Four different types of stainless steel rods were tested and used in the experiment, with base diameters of 1.25, 2.5, 3.75, and 5 mm, each with 5 mm height. In the burn experiment, one rod was transferred to the center of the matrix and kept there for 30 seconds. After that, the rod was removed, producing a central defect area with standard dimensions.

Keratinocytes were cultured on collagen-coated Millicell membranes. Stratified epithelia were formed on the seventh day after the cultures were raised to the air-liquid interface.

**Figure 1:**
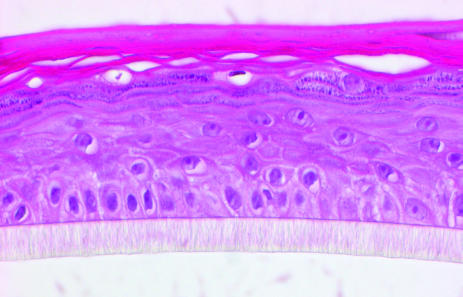
Stratified epithelium produced by 2 × 10^6^ cells seeded.

The seeding was done using fourth passage keratinocytes to enable a higher number of cultured cells, as it was necessary to use 2 × 10^6^ keratinocytes per membrane. In other experiments, 0.5 × 10^6^ and 1 × 10^6^ cells were seeded; however, the resultant epithelia were not uniform. When using 2 × 10^6^ keratinocytes, the epithelium was well developed, uniform, and stratified up to the stratum corneum. This epithelium was developed after 3 days of submerged culture (Figure 1 and Table 1).

This layer was initially cultured in agarose but it did not show good results because the keratinocytes did not attach to the agarose. When fibrin glue was used as a dermal substitute, stratified epithelia developed; however, it was fragile, tearing apart when the culture medium was aspirated.

In the next stage, fibrin gel was used showing good keratinocyte adhesion. However, the gel proved to be too fragile, showing rupture when moved or being aspirated together with the culture medium.

Different volumes of collagen and Matrigel, always in a ratio of 1:1, were tested for the preparation of matrices. The use of 400 μL of collagen and 400 μL of Matrigel was necessary to coat the Millicell membrane. The cross-link was accomplished by adding 80 μL of 0.1% glutaraldehyde, which led to the formation of more stable matrices. In the initial groups, where the cross-link was not performed, some matrices were lost because of gel rupture in the subsequent days.

Once the cultures were raised to the air-liquid interface, the supplementation of the media was carried out by adding culture medium around the matrix. Beginning on the fourth day after seeding, aprotinin was not added to the culture medium without harming the keratinocyte culture. Since the Millicell membrane has pores sized 0.4 μm, the keratinocytes were nourished by imbibition (Table 2).

The burn procedure took place on the seventh day after the cultures were raised to the air-liquid interface or the tenth day after the keratinocyte seeding. Stainless steel rods of different dimensions and masses were evaluated for their burning effects on matrices.

**Figure 2:**
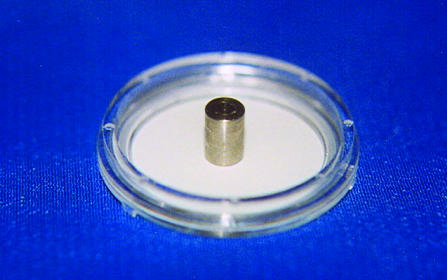
Rod size to produce the burn defect.

It was found that the 12.5-mm^2^ rods became unstable, falling down on the epithelia, and produced irregular burn defects. The 25-mm^2^ rods weighing 0.88 gram produced a circular and uniform central defect, over 4.5% of the epithelium surface, without disrupting the subjacent collagen and Matrigel layer (Figure 2). Some of the 37.5-mm^2^ rods detached the epithelia from the gel when removed after the burning time. All the 50-mm^2^ rods were detached part of the epithelia when removed from the gel contact (Table 3).

Different contact times between the hot metal rods and the epithelia were tested (Table 4).

It was possible to accomplish the burn experiment at 170°C, even with loss of heat to the environment. Different contact times were tested, from 10 seconds to 1 minute. After 10~seconds of heat contact, a complete lesion of the keratinocytes was not achieved. After 60 seconds contact, the heat caused gel softening in the central area. When a gel of collagen and Matrigel was used as a dermal substitute, there was no consistency change even at high temperatures.

## DISCUSSION

Millicell membranes were chosen because they are easy to work with. The Millicell membranes come with a plastic ring that helps grow the epithelia in the air-liquid interface without culture medium flowing out on the keratinocytes.

Cultured keratinocytes were used as a model to avoid the influence of other growth factors produced by fibroblast. As the keratinocytes present a more satisfactory growth in the presence of a dermal substitute, the use of an acellular layer became necessary. This layer was initially cultured in agarose, but it did not show good results because the keratinocytes did not attach to the agarose. When fibrin glue was used as a dermal substitute, stratified epithelia developed; however, it was fragile, tearing apart when the culture medium was aspirated. In the next stage, fibrin gel was used, showing good keratinocytes adhesion. However, the gel proved to be too fragile, showing rupture when moved or being aspirated together with the culture medium. Other authors have cultured keratinocytes on collagen, achieving the development of 6 cell layers.^10^, ^11^ In another study, different dermal substitutes were tested and keratinocytes cultured on a surface with Matrigel showed better adhesion than the ones on other substitutes.^12^

The use of aprotinin in the keratinocyte seeding procedure was a crucial stage to the method development. In the pilot project experiments, collagen degradation by the keratinocytes was observed when the aprotinin was not used. The aprotinin was added to the fibrin, and because of its serine proteinase activity, inhibited the collagen digestion by the keratinocytes.^8^

The seeding was done using fourth passage keratinocytes to enable a higher number of cultured cells, as it was necessary to use 2 × 10^6^ keratinocytes per membrane. When using 2 × 10^6^ keratinocytes, the epithelium was well developed, uniform, and stratified up to the stratum corneum. This epithelium developed after 3 days of submerged culture, findings that were consistent with results of a previous work.^7^

The epithelia were cultured to show terminal differentiation. The aim was to try to simulate as close as possible the same circumstances of an epidermis of a burn patient.

The use of a constant-temperature hot plate and identical stainless steel rods permitted to obtain very similar and easily reproducible burn defects in all matrices. The burning was performed in a similar way as the mechanism of a cutaneous burn. The defect took away the keratinocytes from the central burn area, as it occurs in a third degree burn.

In this study, when rods of different contact surface areas were used, they fell down over the epidermis producing an irregular burn defect. It is important to mention that the proposed model was not intended to simulate an extensive burn surface, but instead to allow the evaluation in vitro of the interaction between keratinocytes and substances or microorganisms introduced into the burn wound.

It was possible to accomplish the burn experiment at 170°C, even with loss of heat to the environment. Different contact times were tested, from 10 seconds to 1 minute. After 10 seconds of heat contact, no loss of keratinocytes was observed. After 60 seconds contact, the heat caused gel softening in the central area. When a gel of collagen and Matrigel was used as a dermal substitute, there was no alteration in physical consistency even at high temperatures. In the first experiments, using agarose gel and later fibrin gel, there was gel liquefaction in the central area due to the high temperature.

The use of isolated keratinocytes as a burn model made possible the analysis of the interactions of these cells with several growth factors involved in the burn process. Thus, it will be possible to perform studies dealing with the wound repairing mechanisms in burn surfaces in a more specific way.

## Figures and Tables

**Table 1:** Number of cells seeded and formation of the epithelium

No. of cells	0.5 × 10^6^	1 × 10^6^	2 × 10^6^	5 × 10^6^
Epithelium	Absent	Absent	Present	Present

**Table 2:** Aprotinin and gel degradation

		
Aprotinin	Present	Absent
Gel degradation	Absent	Present

**Table 3:** Contact area and burn defect produced in the epithelium

Contact area, mm^2^	12.5	25	37.5	50
Burn defect	Irregular	Regular	Irregular	Irregular

**Table 4:** Contact time and burn defect produced

Contact time, s	10	20	30	60
Burn defect	Irregular	Irregular	Regular	Irregular
